# Distinct Patterns of HBV Integration and *TERT* Alterations between in Tumor and Non-Tumor Tissue in Patients with Hepatocellular Carcinoma

**DOI:** 10.3390/ijms22137056

**Published:** 2021-06-30

**Authors:** Jeong-Won Jang, Hye-Seon Kim, Jin-Seoub Kim, Soon-Kyu Lee, Ji-Won Han, Pil-Soo Sung, Si-Hyun Bae, Jong-Young Choi, Seung-Kew Yoon, Dong-Jin Han, Tae-Min Kim, Lewis R. Roberts

**Affiliations:** 1 Division of Gastroenterology and Hepatology, Department of Internal Medicine, College of Medicine, The Catholic University of Korea, Seoul 06591, Korea; blackiqq@catholic.ac.kr (S.-K.L.); tmznjf@catholic.ac.kr (J.-W.H.); pssung@catholic.ac.kr (P.-S.S.); baesh@catholic.ac.kr (S.-H.B.); jychoi@catholic.ac.kr (J.-Y.C.); yoonsk@catholic.ac.kr (S.-K.Y.); 2 The Catholic University Liver Research Center, Department of Biomedicine & Health Sciences, College of Medicine, The Catholic University of Korea, Seoul 06591, Korea; clover@catholic.ac.kr (H.-S.K.); topiary@catholic.ac.kr (J.-S.K.); 3 Department of Biomedicine & Health Sciences, Graduate School, The Catholic University of Korea, Seoul 06591, Korea; d88020@catholic.ac.kr; 4 Department of Medical Informatics, Cancer Research Institute, College of Medicine, The Catholic University of Korea, Seoul 06591, Korea; tmkim@catholic.ac.kr; 5 Division of Gastroenterology and Hepatology, Mayo Clinic College of Medicine and Science, Rochester, MN 55902, USA; Roberts.Lewis@mayo.edu

**Keywords:** hepatitis B virus, virus integration, liver cancer, telomerase, point mutation

## Abstract

Although hepatitis B virus (HBV) integration into the cellular genome is well known in HCC (hepatocellular carcinoma) patients, its biological role still remains uncertain. This study investigated the patterns of HBV integration and correlated them with *TERT* (telomerase reverse transcriptase) alterations in paired tumor and non-tumor tissues. Compared to those in non-tumors, tumoral integrations occurred less frequently but with higher read counts and were more preferentially observed in genic regions with significant enrichment of integration into promoters. In HBV-related tumors, *TERT* promoter was identified as the most frequent site (38.5% (10/26)) of HBV integration. *TERT* promoter mutation was observed only in tumors (24.2% (8/33)), but not in non-tumors. Only 3.00% (34/1133) of HBV integration sites were shared between tumors and non-tumors. Within the HBV genome, HBV breakpoints were distributed preferentially in the 3’ end of HBx, with more tumoral integrations detected in the preS/S region. The major genes that were recurrently affected by HBV integration included *TERT* and *MLL4* for tumors and *FN1* for non-tumors. Functional enrichment analysis of tumoral genes with integrations showed enrichment of cancer-associated genes. The patterns and functions of HBV integration are distinct between tumors and non-tumors. Tumoral integration is often enriched into both human-virus regions with oncogenic regulatory function. The characteristic genomic features of HBV integration together with *TERT* alteration may dysregulate the affected gene function, thereby contributing to hepatocarcinogenesis.

## 1. Introduction

Chronic infection with hepatitis B virus (HBV) is a leading cause of liver-related morbidity and mortality, especially in Asia and Africa. Approximately 300 million people worldwide are estimated to be chronically infected with HBV. This virus is also the major cause of hepatocellular carcinoma (HCC). During long-lasting host–virus interaction, immune-mediated cytolysis may result in fibrosis and eventually, cirrhosis as a key risk factor for HCC. On the other hand, the production of the onco-proteins, such as HBx, L-HBs, or MHBst, and integration of HBV DNA into the human genome are also important drivers of liver carcinogenesis, having direct oncogenic potential [1].

HBV integration was first reported in the early 1980s [2]. Although the integration event is not essential for viral replication, HBV DNA integration can contribute to liver carcinogenesis by inducing genomic instability and altering expression of cancer-related genes [3]. Recent advances in massive parallel-sequencing technology have enabled genome-wide surveys of HBV integration sites throughout the human genome. Telomerase reverse transcriptase (*TERT*) was reported to be among the most frequent sites integrated by HBV [4,5].

Telomerase is a ribonucleoprotein polymerase that is responsible for maintaining chromosomal integrity and stability. In normal somatic cells, telomerase is repressed, and hepatocytes under chronic inflammation undergo telomere shortening, which can lead to chromosomal end-to-end fusion and instability that promote cellular senescence and apoptosis [6]. However, during hepatocarcinogenesis, some cells can overcome the senescence by telomere maintenance via telomerase reactivation [7]. More than 90% of HCCs display telomerase reactivation, which is associated with *TERT* promoter mutations, *TERT* amplification, *TERT* translocation, and HBV-*TERT* integration [6,8]. Telomere dysfunction and *TERT* are tightly linked to hepatocarcinogenesis.

Recent studies using next-generation sequencing (NGS) have yielded a list of recurrently mutated genes in HCC, including *TERT* (telomere maintenance), *TP53* (cell-cycle pathway), *CTNNB1/AXIN1* (*WNT/β-catenin* pathway), *ARID1A/ARID2* (epigenetic modifier), and *NFE2L2* (oxidative stress pathway) [5,9,10]. Among these, *TERT* promoter mutations are the most frequently identified in HCC tissues and progressively increase in frequency from premalignant lesions to early HCC, supporting the notion that this may be a gatekeeper mutation in hepatocarcinogenesis [11,12]. Despite this emerging data, the clinical implications of HBV integration and its relationship with *TERT* alterations in driving hepatocarcinogenesis are incompletely understood.

In this study, we present a survey of HBV integration in paired samples from HCC patients undergoing surgical treatment. Through analysis, we identified distinct patterns of HBV integration and *TERT* alterations between tumor and non-tumor tissues as well as the biological functions of genes with HBV integrations in HCC.

## 2. Results

### 2.1. Patient Characteristics

We analyzed 66 paired tumor and non-tumor samples from 33 patients with HCC. The subjects were 29 male and 4 female patients, aged 52.6 ± 10.2 years. Most of the patients had HBV-associated HCC (*n* = 26; 78.8%) and Child-Pugh class A liver function (*n* = 29; 87.9%). Tumor size was 4.1 ± 2.0 cm, and tumor stage was Barcelona Clinic Liver Cancer stage-A in 20 (60.6%) and -B in 10 (30.3%) patients. All of the patients underwent surgical therapy of either hepatectomy (*n* = 25) or liver transplantation (LT) (*n* = 8). The clinical characteristics of the patients are shown in Table 1.

### 2.2. Detection and Validation of Hepatitis B Virus (HBV) Integration Breakpoints

Using probe-based HBV capture technology, a total of 1133 HBV integration breakpoints were observed within these 66 samples, with a range of 1 to 107 breakpoints per sample. Overall, HBV integration was detected in all (100%, 26/26) HBV-positive non-tumor samples, and in 84.6% (22/26) of HBV-related tumor samples. Interestingly, HBV integration was also observed in a subset of patients with HCV infection (20%, 1/5) or non-viral diseases (50%, 1/2). These two patients had IgG anti-HBc, which is a serologic marker for remote past HBV infection. To confirm the HBV integrations, we randomly selected 20 breakpoints at the affected genes for polymerase chain reaction (PCR) analysis in the present and other independent samples and successfully validated 90.0% (18/20) of the integration sites (Supplementary Table S1).

### 2.3. Genomic Locations of HBV Integration Breakpoints

**Figure 1:**
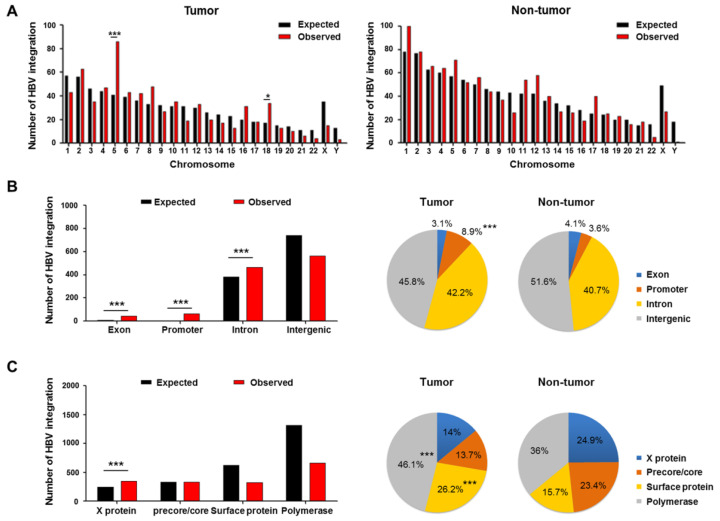
(**A**) Distribution of hepatitis B virus (HBV) integration breakpoints across all human chromosomes. (**B**) The proportion of HBV integration sites in genic and intergenic areas in paired tumor and non-tumor tissues. (**C**) HBV integration sites in the HBV genome. HBV integration breakpoints were counted by allowing overlaps between the four open reading frames. *p*-value (binomial distribution): *** < 0.001.

**Figure 2:**
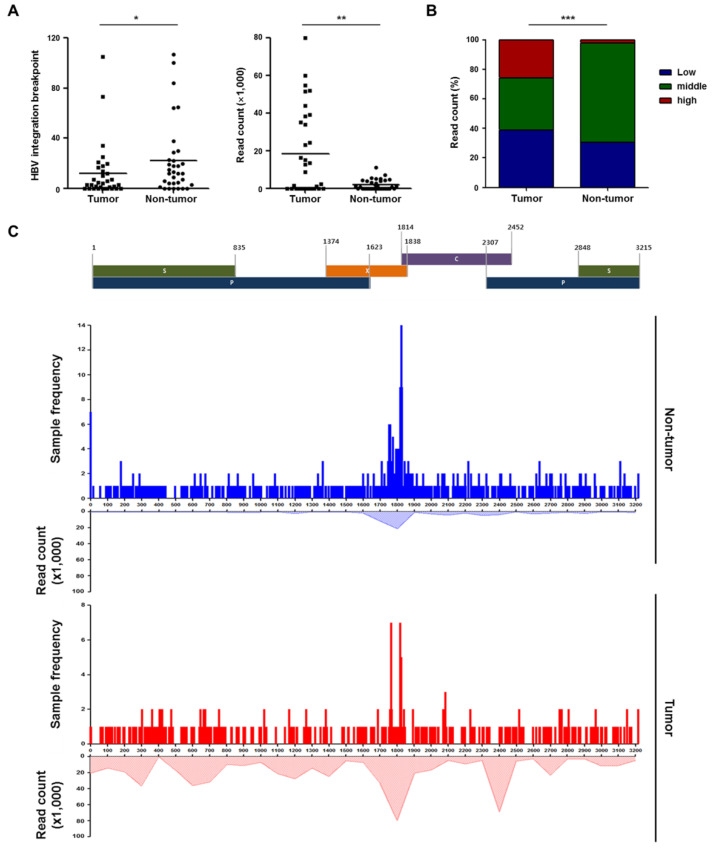
Comparison of HBV integrations between paired tumor and non-tumor tissues. (**A**) HBV breakpoints and read counts. (**B**) Proportion of tissues with low, middle, and high read counts. (**C**) Location, frequency, and read counts of HBV breakpoints in the HBV genome of tumors and non-tumor tissues. *p*-value (binomial distribution): * < 0.05, ** < 0.01, *** < 0.001.

Figure 1A shows HBV integration breakpoints distributed across the entire human genome. HBV was preferentially integrated into chromosomes 5 and 18 (all *p* < 0.05) in tumors, as compared to the expected result. Although not statistically significant, there were preferential integrations of HBV into genic regions (defined as the combination of promoters (5 kb upstream of transcription start site), exons (including 3′-untranslated region) and introns) in tumors but not non-tumor tissues (54.2% (213/393) vs. 48.4% (358/740), respectively; *p* = 0.062). In particular, there was specific enrichment of HBV integrations into the promoter region of genes in tumors versus non-tumor tissues (Figure 1B). The enrichments of HBV integration might be associated with recurrent integrations into *TERT*, *MLL4*, or other sites. Notably, despite the fewer tumoral integration breakpoints (Figure 2A), the absolute number of HBV-promoter integrations was higher in tumors than in adjacent non-tumor tissues (8.9% (35/393) vs. 3.6% (27/740), respectively; *p* < 0.001). For the HBV genome, integration was more frequently found in the HBx (X protein), but less frequently found in the polymerase as compared to the expected frequencies (Figure 1C). HBV integration into the preS/S region (surface protein) was more frequently observed in tumors versus non-tumor tissues (*p* < 0.001).

### 2.4. Patterns of HBV Integration Breakpoints in Tumors and Non-Tumors

The number of HBV integration breakpoints was significantly higher in adjacent tissues than in tumor tissues, with average breakpoints of 22.42 and 11.91 per sample, respectively (*p* = 0.015), but the total read count was significantly higher in tumors than in non-tumor tissues (18,483 vs. 2178 per sample, respectively; *p* = 0.001) (Figure 2A; Supplementary Figure S1). The proportion of tissues with low (≤10), middle (>10−1000), and high (>1000) chimeric read counts was significantly different between the tumor and non-tumor samples. In particular, the proportion of HBV breakpoints with a high chimeric read count (>1000) was significantly greater in tumors than in non-tumor tissues (*p* < 0.001; Figure 2B).

For both tumors and non-tumor tissues, 31.3% (355/1133) of all the breakpoints (19.6% (77/393) in tumors and 37.6% (278/740) in non-tumors) were preferentially mapped within the nt 1700–1999 region of the HBV genome that encompasses various functional sequences (Figure 2C). However, the junction patterns observed across the HBV genome were quite distinct between tumors and non-tumor tissues. Compared to the non-tumor tissues, tumoral integration breakpoints revealed a lower frequency of junctions across the HBV genome but a higher depth of read counts (Figure 2C). Overall, there was a distinct difference in the pattern of HBV integration breakpoints between tumors and non-tumor tissues (Figure 2), which suggests that clonal expansion occurs in the HCCs rather than in the non-HCC tissues.

### 2.5. Recurrent HBV Integration Sites

**Figure 3:**
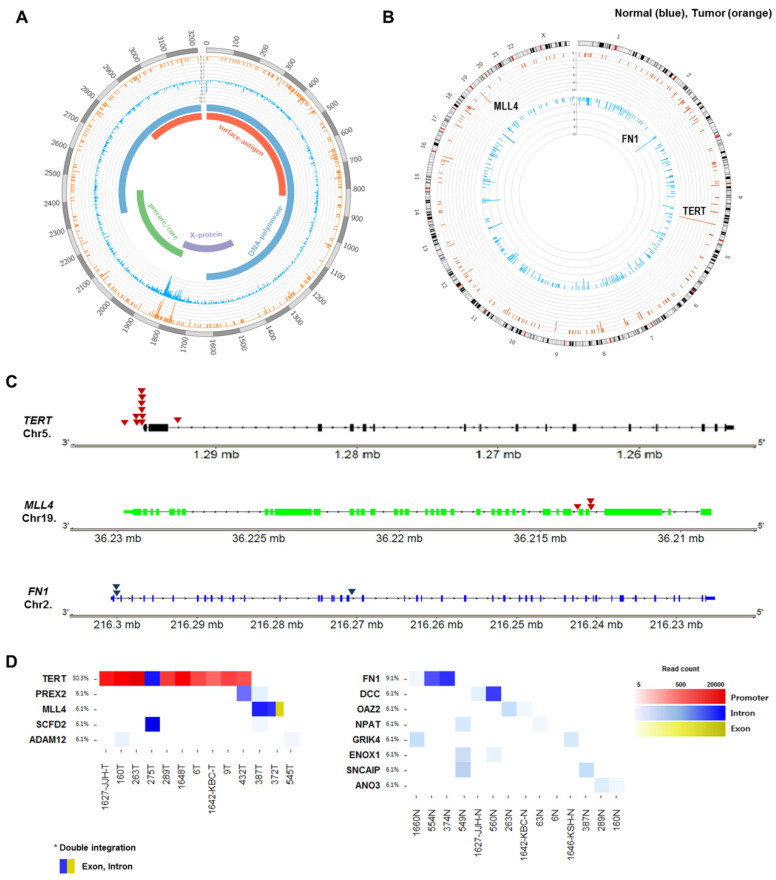
Circos plot showing the distribution of integration sites in the (**A**) HBV genome and (**B**) human genome. Bars in orange and sky blue indicate HBV integration breakpoints in tumor and adjacent non-tumor tissues, respectively. (**C**) The genomic locations of the three high-frequency HBV-integrated genes (*TERT*, *MLL4*, and *FN1*). (**D**) Intragenic locations of all recurrent target genes of HBV integration in tumors (T) and non-tumor (NT) tissues. *TERT*, telomerase reverse transcriptase; *MLL4*, mixed lineage leukemia 4; *FN1*, fibronectin 1; *PREX2*, phosphatidylinositol-3,4,5-trisphosphate dependent Rac exchange factor 2; *SCFD2*, sec1 family domain containing 2; *ADAM12*, ADAM metallopeptidase domain 12; *DCC*, DCC netrin 1 receptor; *OAZ2*, ornithine decarboxylase antizyme 2; *NPAT*, nuclear protein, coactivator of histone transcription; *GRIK4*, glutamate ionotropic receptor kainate type subunit 4; *ENOX1*, Ecto-NOX disulfide-thiol exchanger 1; *SNCAIP*, synuclein alpha interacting protein; *ANO3*, anoctamin 3.

The distribution of HBV integration breakpoints in the HBV and human genomes was depicted in circos plots (Figure 3A,B). Only 3.00% (34/1133) of HBV-integrated human genomic sites were shared between tumors and non-tumors, again indicating the different features of integration in tumors and non-tumor tissues.

In tumors, the *TERT* promoter was the most common site affected by HBV integration (Figure 3B). For HBV-related HCC, five genes were recurrently integrated by HBV, including *TERT* (38.5%, 10/26), *MLL4* (11.5%, 3/26), *ADAM12* (7.7%, 2/26), *PREX2* (7.7%, 2/26), and *SCFD2* (7.7%, 2/26) (Table 2). In contrast to tumors, the adjacent non-tumor tissues harbored eight genes with recurrent HBV integration, including *FN1*, *DCC*, *OAZ2*, *ANO3*, *ENOX1*, *GRIK4*, *NPAT*, and *SNCAIP.*

**Figure 4:**
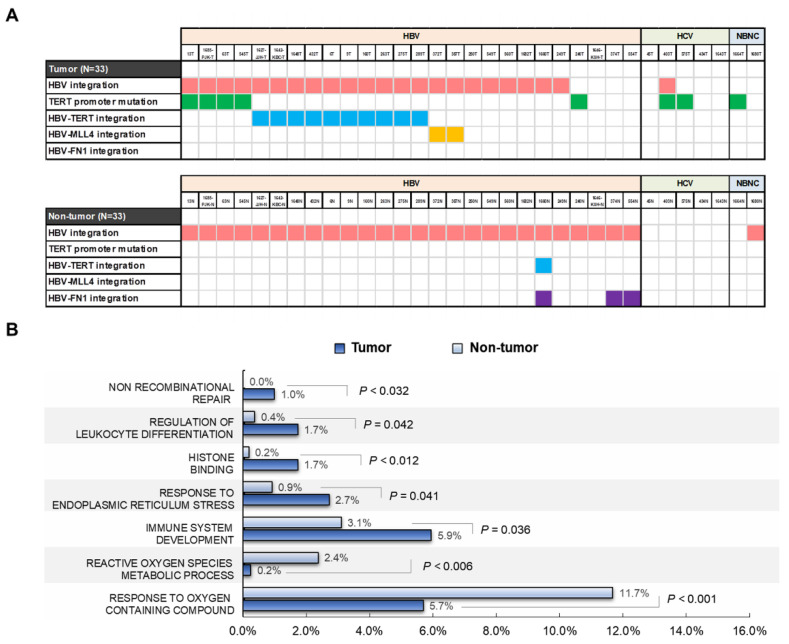
(**A**) Schematic view of overall HBV integrations, 124 C > T mutations in the *TERT* promoter, HBV-*TERT*, HBV-*MLL4*, and HBV-*FN1* integrations in the paired HCC (upper panel) and non-HCC tissues (lower panel). (**B**) Functional enrichment analysis of genes affected by HBV integration in tumors versus non-tumor tissues.

The frequency of recurrent HBV integration into *TERT* or *MLL4* was particularly high in tumor samples, accounting for 54.5% (12/22) of HBV-related HCC with integrations. However, the HBV-*TERT* and HBV-*MLL4* integrations were mutually exclusive (Figure 4A). Interestingly, as a recurrent integration, we noticed HBV-*FN1* integration in only HBV-associated non-tumor (11.5%; 3/26) samples. The genomic locations of the three high-frequency HBV-integrated genes are mapped in Figure 3C. Intragenic locations of all recurrently targeted genes with HBV integration in the tumors and non-tumor tissues are shown in Figure 3D.

### 2.6. Telomerase Reverse Transcriptase (TERT) Promoter Hot Spot Mutation

Next, we evaluated the *TERT* promoter mutation known as a gatekeeper mutation in HCC within the 66 samples. The *TERT* mutations were observed only in tumors but not in non-tumors (24.2% (8/33) vs. 0.0% (0/33); *p* = 0.005) (Figure 4A). Among previously known mutations in the promoter of *TERT*, we found only the −124C > T (100%), but detected no specific mutation in the adjacent non-HCC tissues. The average number of breakpoints in tumor samples with and without *TERT* mutations was 7.6 and 12.8, respectively. The corresponding number within HBV-related HCCs was 12.0 and 14.6, respectively. The number of HBV breakpoints was higher in samples without *TERT* mutations than in those with *TERT* mutations, but this difference did not reach statistical significance (Supplementary Figure S2). HBV-*TERT* integration was mutually exclusive with *TERT* promoter mutations (Figure 4A).

### 2.7. Functional Enrichment Analysis of HBV-Integrated Genes

Gene ontology analysis of the 225 non-redundant integrated genes from the tumor tissues showed that they were significantly enriched in terms related to non-recombinational repair, regulation of leukocyte differentiation, histone binding, response to endoplasmic reticulum stress, and immune system development (*p* < 0.05). In the annotation of non-tumoral genes, the 383 integrated genes were significantly enriched in the reactive oxygen species metabolic process and response to oxygen-containing compounds (*p* < 0.05; Figure 4B). In the analysis of gene function, the tumoral genes with recurrent integrations all appeared to be related to carcinogenesis, including cell aging and immortalization, transcriptional regulation, development and cell-to-cell interaction, protein transport, and cellular signaling pathways (Supplementary Table S2).

## 3. Discussion

Our analysis demonstrated distinctly different patterns of HBV integrations between tumors and non-tumor tissues. Despite having fewer breakpoints, tumors exhibited a higher proportion of HBV integration with high chimeric read counts as well as promoter integration. The absolute number of genes with recurrent HBV integration was significantly lower in tumors than in non-tumor tissues, but the recurrent integrations in tumors were enriched in regions of cancer-associated genes. In particular, the *TERT* promoter was identified as the most frequent site of HBV integration in tumors. For the HBV genome, HBV breakpoints were distributed preferentially in the 3’ end of HBx, with more tumoral integrations into the preS/S region, which encompass viral transcriptional regulators. The overall findings indicate that HBV integration events in tumors are often enriched into both host and virus regions with regulatory functions, which may cause the dysregulation of the affected gene function, thereby contributing to carcinogenesis.

HBV integrations into the cellular genome reportedly occur randomly, early after infection [13]. However, unlike non-tumoral integrations involving more breakpoints with lower read counts, tumoral integrations exhibited fewer breakpoints with higher read counts. When analyzing the patterns of HBV genomic locations, non-tumor tissues showed a higher frequency of integration, which were dispersed across the HBV genome. In contrast, tumors showed less frequent breakpoints across the HBV genome, but more areas with high-depth read counts, which are very rarely observed in non-tumor tissues. In addition, tumoral integrations were more enriched in cancer-associated genes. The distinct pattern of HBV integration implies that there is an increased burden of cancer cells with viral integrations into oncogenic genes generated through clonal expansion, whereas in non-tumor tissues, HBV integrations appear to be random or non-clonal without conferring a survival advantage.

Our analysis identified several genes with recurrent HBV integration, including *TERT*, *MLL4*, *ADAM12*, *PREX2*, and *SCFD2* in tumor tissues. These genes can play carcinogenic roles, such as in cellular immortalization, transcriptional regulation, development, cell-to-cell interaction, protein transport, and cell signaling. In particular, the preferential integration into *TERT* or *MLL4* in HBV-associated HCCs suggests the active involvement of HBV integration in the major oncogenic pathways of HCC, such as telomere stability and chromatin remodeling [11]. Our observation of the most-often recurring integration sites such as *TERT* and *MLL4* in tumors as well as *FN1* in non-tumor tissues is compatible with previous reports [4,14]. We also demonstrated several novel recurring integration genes, such as *ADAM12*, *PREX2*, and *SCFD2*. It is noteworthy that these genes with recurrent integrations in tumors are not shared with integration-recurring genes in non-tumor tissues, again indicating that tumoral integration patterns are different from those found in non-tumor samples.

Importantly, our analysis revealed the implications of HBV integration and *TERT* promoter mutation, which was recently suggested as a potential biomarker in hepatocarcinogenesis [6,15]. We found HBV integration in 84.6% (22/26) of HBV-related tumors, with 38.5% (10/26) containing HBV insertions into the *TERT* locus. *TERT* promoter mutations were observed only in HCC, not in non-HCC tissues. Although HBV-*TERT* integrations and *TERT* promoter mutations were mutually exclusive [5], the presence of either HBV integration or *TERT* mutations was associated with the increased transcriptional activation of *TERT* [4,16,17]. Interestingly, *MLL4*, another recurrently targeted gene, also occurred mutually exclusively with *TERT* promoter mutations or HBV-*TERT* integrations (Figure 4A). Although the mechanisms remain unclear, the mutually exclusive genomic events may suggest redundancy underlying their functionality [6] or imply that the acquisition of either type of the *TERT* alterations might be sufficient to result in activation of *TERT* in HCC [18]. Either *TERT* promoter mutations or HBV integration into the two genes occurred in 65.4% (17/26) of HBV-related HCCs in our study, indicating a crucial role of *TERT* or *MLL4* genetic alterations in HBV-associated hepatocarcinogenesis.

Of note, viral breakpoints were most preferentially observed in the area of nt 1700–1999 of the HBV genome. The HBx and precore/core regions harbor multiple functional sequences, such as viral enhancer and basal core promoter regions. It was reported that upon integration, the 3′ end of the HBx gene is often deleted, and HBx–human chimeric transcripts that translate to chimeric proteins are commonly observed [19]. HBV insertion can also produce mutant HBV proteins such as truncated X or preS/S proteins, which may transactivate signaling pathways implicated in tumorigenesis [1,20]. Thus, this genomic structural preference for viral breakpoints in the HBV genome may impose translational dysregulation of the affected genes through cis-regulatory effects as well as facilitate HBV inserts to form oncogenic proteins [4].

Overall, our analysis of paired tissues suggests that tumoral integrations are not totally random, most frequently affecting actively transcribed, gene-dense regions and regulatory areas such as promoters [21]. The observation of the preferential enrichment of tumoral HBV breakpoints in promoter regions suggests their role in regulating the function of the affected genes. The *TERT* promoter and HBV PreS/S or X integrants were the most common integration breakpoints for the cellular and viral genomic sites, respectively. The characteristic genomic features may help induce altered expression or transcriptional activation of cancer-related genes by insertion of HBV enhancer within integrated HBV sequences in tumor cells [16]. Indeed, it has been reported that insertion of viral enhancers may trigger promoter activation independently of position and orientation [22].

Our analysis was performed only on a limited number of patients. Our results may not be generalized to other settings, since the samples were significantly skewed towards male subjects (87.9%). Although read counts ≥5 were herein taken as breakpoints, we have often experimentally confirmed HBV integrated molecules even with counts of less than 5. The exact cut-off for read counts as a true signal remains to be further explored. Etiologies and treatments of HCC were also heterogeneous, including hepatectomy and LT. Thus, we could not correlate HBV integrations with clinical outcomes. Nevertheless, unlike previous studies that involved only tumors or non-paired tissues, our study has strengths in that it evaluated paired tumors and the matched non-tumor tissues, and thus would facilitate the direct and reliable comparison of the roles of HBV integration between tumors and non-tumors.

## 4. Materials and Methods

### 4.1. Patients and Samples

We obtained 33 pairs of HCCs and their matched non-HCC liver tissues via liver surgery from patients with HCC at The Catholic University of Korea, Seoul, South Korea, between October 2016 and December 2017. All patients were diagnosed with HCC, which was histologically confirmed in surgical specimens. The samples were unaffected by hemangioma or other benign tumors and were immediately frozen in liquid nitrogen and then stored at −80 °C. This study was approved by the Ethics Committee of The Catholic University of Korea, and written informed consent was obtained from all patients.

### 4.2. Preparation of HBV Probes

Probe-based HBV capture followed by NGS technology was performed to survey HBV integration in HCC (Supplementary Figure S3). The probes for HBV hybridization were designed to tile based on the eight Korean full HBV genome sequences (GenBank Accession numbers AY641559.1, DQ683578.1, GQ872211.1, GQ872210.1, JN315779.1, KR184660.1, AB014381.1, AB014395.1, and D23680.1 Hepatitis B virus complete genome sequence (https://www.ncbi.nlm.nih.gov/nuccore, accessed on 2 March 2018), as described elsewhere [23]. We designed 215 probes to span the entire HBV genome. The probe group size was 25.595 kbp. With these designed probes, we achieved 100% sequence coverage for all given HBV sequences. 

### 4.3. Enrichment of HBV-Integrated Fragments and Capture Sequencing 

The Illumina NGS workflow we used to capture the integrated HBV sequences was described elsewhere [23]. Briefly, 1 ug of genomic DNA was fragmented by adaptive focused acoustic technology (AFA; Covaris) and then repaired; an ‘A’ was ligated to the 3’ end, Agilent adaptors were ligated to the fragments, and the adaptor-ligated product was PCR-amplified. We captured HBV using 250 ng of DNA library according to the standard Agilent SureSelect Target Enrichment protocol. Hybridization of capture bait was performed at 65 °C using a heated cycler lid option at 105 °C for 24 h. The purified product was quantified according to the manufacturer’s instructions (qPCR quantification protocol guide) and qualified using the TapeStation DNA screentape D1000 (Agilent). Finally, we did paired-end 100-bp read-length sequencing of the purified captured DNAs by Illumina HiSeq 2500 (Illumina, San Diego, CA, USA) following the manufacturer’s instructions.

### 4.4. Detection of the HBV-Human Chimeric Reads

The identification of the HBV-human chimeric reads was described previously [23]. Briefly, a modified reference was generated by merging the human (UCSC assembly hg19, original GRCh37 from National Center for Biotechnology Information (NCBI), February 2009) and HBV (DQ683578.1) genome. The paired-end reads were mapped to the reference by Burrows-Wheeler Aligner (BWA-MEM) (bwa-0.7.12) and then chimeric reads were extracted using an in-house script and break points were predicted from chimeric reads aligned to both the human and the HBV genome. In our study, we used mapping quality (MQ) and counts of host-virus chimeric DNA fragments for HBV integration breakpoint calling (Supplementary Table S3). For quality of phred score, MQ cut-off values of 10, 20, 30, and 40 indicate 10%, 1.0%, 0.1% and 0.01% probability of incorrect base call, respectively. We defined HBV breakpoints with a chimeric read count ≥5 and average MQ ≥20 as true signal.

### 4.5. PCR-Based Sanger Sequencing for Validation 

PCR and Sanger sequencing were done to validate integrated HBV in the 20 randomly selected HBV-human junction breakpoints at the *TERT*, *MLL4*, and *FN1* genes (Supplementary Figure S4). Sequencing primers were designed based on the paired-end reads, with one primer located in the human genome and the other in the HBV genome. The PCR conditions and primer sequences for the cases are shown in Supplementary Table S4.

### 4.6. Sequencing of the TERT Promoter Region 

The promoter region of *TERT* covering the previously known hotspot mutations (-124C>T, -146C>T) was PCR-amplified as previously described [4]. Briefly, genomic DNA was extracted from liver tissue using InstaGene Matrix (Bio-rad, Hercules, CA, USA) and MG Tissue SV (Doctor Protein Inc., Geumchun-gu, Korea). The PCR was performed with the following primer pairs: forward, 5’-CTGGCGTCCCTGCACCCTGG-3’ and reverse, 5’-ACGAACGTGGCCAGCGGCAG-3’, and the Dr. MAX DNA polymerase (Doctor Protein Inc., Geumchun-gu, Korea) in the following conditions: 95 °C for 5 min; 95 °C for 30 s; 62 °C for 30 sec; 72 °C for 1 min; 35 cycles; and 72 °C for 10 min.

### 4.7. Functional Enrichment Analysis of HBV-Integrated Genes

For gene functional enrichment analysis, we collected Hallmark (H), C2 (curated), and C5 (Gene Ontology) functional gene sets as available in the MSigDB database (http://www.gsea-msigdb.org/gsea/msigdb/index.jsp, v7.2, accessed on 10 May 2019). The significance of enrichment for non-tumor and tumor HBV-integrated genes in individual gene sets was estimated by Fisher’s exact test.

### 4.8. Statistical Analysis

Variables were expressed as mean ± standard deviation or median (range). Categorical variables were compared with the chi-square test or the Fisher exact test. Means and medians were analyzed by the Student *t* test and Wilcoxon rank-sum test, respectively. A 2-sided *p* value less than 0.05 was considered to be statistically significant. All statistical analyses were performed using SPSS version 20 software (IBM Corp., Armonk, NY, USA). 

## 5. Conclusions

In conclusion, this study shows the distinct patterns of HBV integration between tumors and non-tumor tissues. Tumoral integrations involve specific enrichment of genic regions, particularly promoters, and clonal expansion of integrated cells, whereas non-tumor integrations show diffuse, high-frequency distribution with low-depth read counts, suggesting more random integration events. The *TERT* promoter was the most frequent integration site, and its mutation was exclusively observed in tumors. Tumoral integrated genes were functionally enriched in oncogenic pathways, with the recurrent targets of integration all being cancer-associated genes. Our survey clearly reveals the characteristic signatures of genomic features driven by HBV integration in tumors and improves the understanding of the biological function of HBV integration during hepatocarcinogenesis.

## Figures and Tables

**Table 1:** Baseline characteristics of the enrolled patients.

Characteristics	HCC (*n* = 33)
Sex (male/female, %)	29 (87.9)/4 (12.1)
Age (years)	52.6 ± 10.2 (29–72)
Etiology (HBV/HCV/NBNC, %)	26 (78.8)/5 (15.2)/2 (6.1)
AST (U/L)	54 (16−97)
ALT (U/L)	36 (14−73)
Total bilirubin (mg/dL)	0.8 (0.2–29.9)
Albumin (g/dL)	3.8 ± 0.5
PT (INR)	1.2 ± 0.2
Child-Pugh class (A/B/C, %)	29 (87.9)/3 (9.1)/1 (3.0)
Tumor size (cm)	4.1 ± 2.0
Tumor number (single/multiple, %)	27 (81.8)/6 (28.2)
α-fetoprotein (ng/mL)	71.3 (1.7–59403)
BCLC stage (A/B/C, %)	20 (60.6)/10 (30.3)/3 (9.1)
Treatment (resection/LT)	25 (75.8)/8 (24.2)

Data are expressed as mean ± SD or median (range). Data are presented as the *n* (%) for categorical variables, unless otherwise indicated. HCC, hepatocellular carcinoma; HBV, hepatitis B virus; HCV, hepatitis C virus; NBNC, non-HBV non-HCV; AST, aspartate aminotransferase; ALT, alanine aminotransferase; PT, prothrombin time; INR, international normalized ratio; BCLC, Barcelona Clinic Liver Cancer.

**Table 2:** Recurrently targeted genes with HBV integration in HCC.

Gene Name	Chr	Location	HBV Proteins	Samples
*TERT*	5	Promoter	X protein	9T, 1648T
	5	Promoter	X protein, Precore/core protein	1627-JJH-T, 263T
	5	Promoter	Precore/core protein	289T, 432T, 1648T
	5	Promoter	Polymerase, Surface antigen	1642-KBC-T, 6T, 160T
	5	Promoter	Polymerase	6T, 9T, 289T, 432T
	5	Intron	Polymerase, Surface antigen	275T
*MLL4*	19	Intron	Polymerase, X protein	372T, 387T
	19	Exon	Polymerase, surface antigen	372T
	19	Intron	Polymerase	387T
*PREX2*	8	Intron	Precore/core protein	387T
	8	Intron	Polymerase, X protein	432T
*SCFD2*	4	Intron	Polymerase	275T
	4	Intron	X protein	387T
*ADAM12*	10	Intron	Polymerase, Surface antigen	160T
	10	Intron	Polymerase	545T

Chr: chromosome.

## Data Availability

The data presented in this study are available on request from the corresponding author. Associated clinical data cannot be provided to maintain patient confidentiality.

## Supplementary Materials

The following are available online at https://www.mdpi.com/article/10.3390/ijms22137056/s1, Figure S1: HBV integration breakpoints and read counts in tumor and matched normal tissues of each patient, Figure S2: The number of HBV integration breakpoint in tumors with and without *TERT* mutation, Figure S3: The schematic view of probe-based HBV capture assay followed by next-generation sequencing (NGS) technology for detecting HBV integration, Figure S4: Sanger sequencing for validation of HBV integration, Table S1: Validation results for the HBV integration sites, Table S2: Genes with recurrent HBV integrations, Table S3: Mapping quality (MQ) and base call accuracy, Table S4: Polymerase chain reaction (PCR) condition and primer design for validation.

## Abbreviations

ADAM12	ADAM metallopeptidase domain 12
AFP	alpha-fetoprotein
ALT	alanine aminotransferase
ANO3	anoctamin 3
BCLC	Barcelona Clinic Liver Cancer
BWA-MEM	Burrows-Wheeler Aligner
CH	chronic hepatitis
CHB	chronic hepatitis B
Chr	chromosome
DCC	DCC netrin 1 receptor
ENOX1	Ecto-NOX disulfide-thiol exchanger 1
GRIK4	glutamate ionotropic receptor kainate type subunit 4
HBsAg	hepatitis B surface antigen
HBV	hepatitis B virus
HCC	hepatocellular carcinoma
HCV	hepatitis C virus
INR	international normalized ratio
LC	liver cirrhosis
MLL4	mixed lineage leukemia 4
NBNC	non-HBV non-HCV
NCBI	National Center for Biotechnology Information
NPAT	nuclear protein, coactivator of histone transcription
OAZ2	ornithine decarboxylase antizyme 2
PREX2	phosphatidylinositol-3,4,5-trisphosphate dependent Rac exchange factor 2
PT	prothrombin time
SCFD2	sec1 family domain containing 2
SNCAIP	synuclein alpha interacting protein
TERT	telomerase reverse transcriptase)
