# Supplementary material for: Distinct Patterns of HBV Integration and TERT Alterations between in Tumor and Non-Tumor Tissue in Patients with Hepatocellular Carcinoma
Source: Int J Mol Sci. 2021 Jun 30;22(13):7056. doi: 10.3390/ijms22137056 (PMC8268258; doi:10.3390/ijms22137056)
Supplement: Supplementary file 1 [file ijms-22-07056-s001.zip › ijms-1257134-supplementary.pdf]

Supplementary Figure S1. HBV integration breakpoints and read counts in tumor and matched normal tissues of each patient

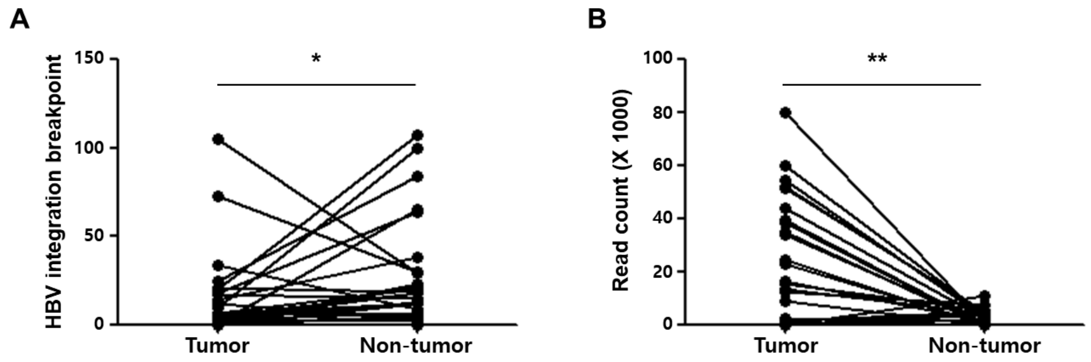

(**A**) The number of HBV integration breakpoints in each patient (P = 0.015). (**B**) Read counts of HBV integration in each patient (P = 0.001). Solid lines connect tumors and their matched non-tumor samples for each patient.

Supplementary Figure S2. The number of HBV integration breakpoint in tumors with and without TERT mutation

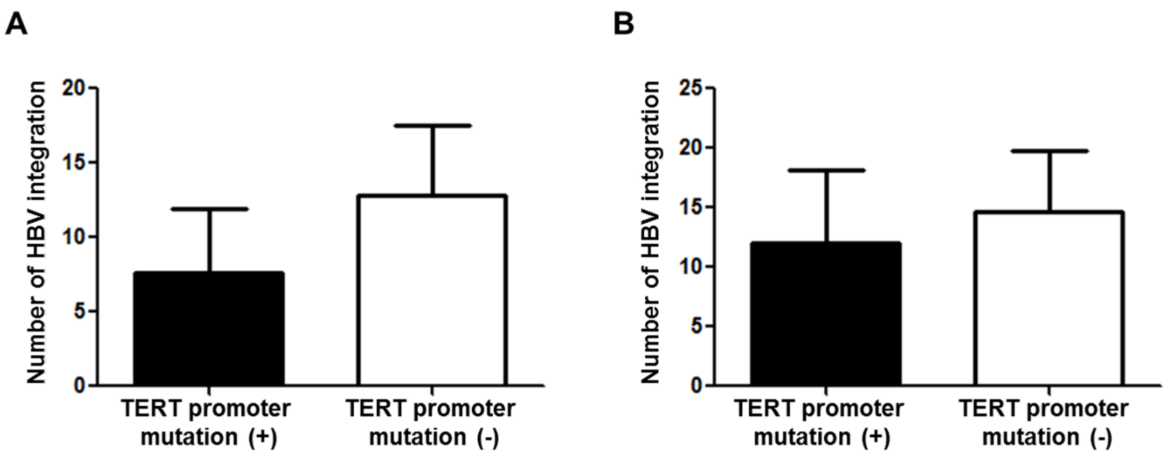

(**A**) The number of HBV integration breakpoint in all the 33 tumors (P = 0.548). (**B**) The number of HBV integration breakpoint in HBV-related tumors (P = 0.821).

Supplementary Figure S3. The schematic view of probe-based HBV capture assay followed by NGS technology for detecting HBV integration

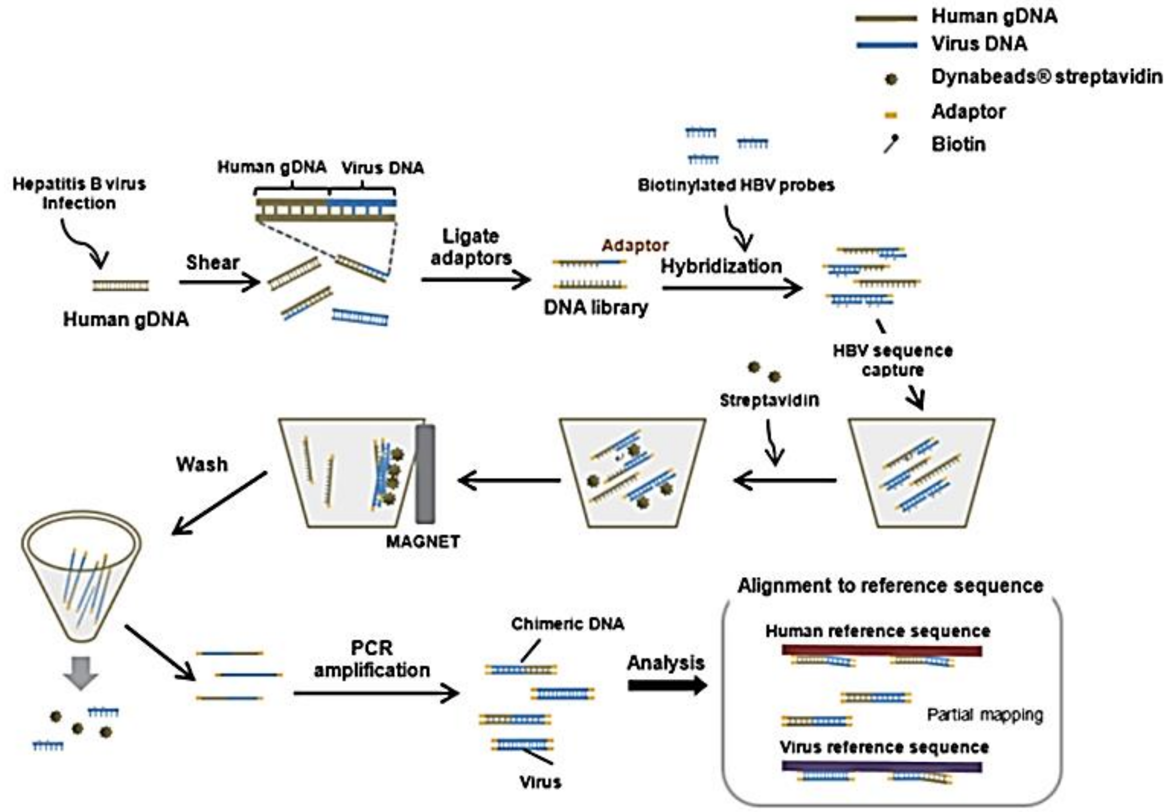

Reprinted from "Persistence of intrahepatic hepatitis B virus DNA integration in patients developing hepatocellular carcinoma after hepatitis B surface antigen seroclearance", by Jang et al, 2021, Clin Mol Hepatol, 27, 207-218.

NGS, next-generation sequencing

Supplementary Figure S4. Sanger sequencing for validation of HBV integration

A

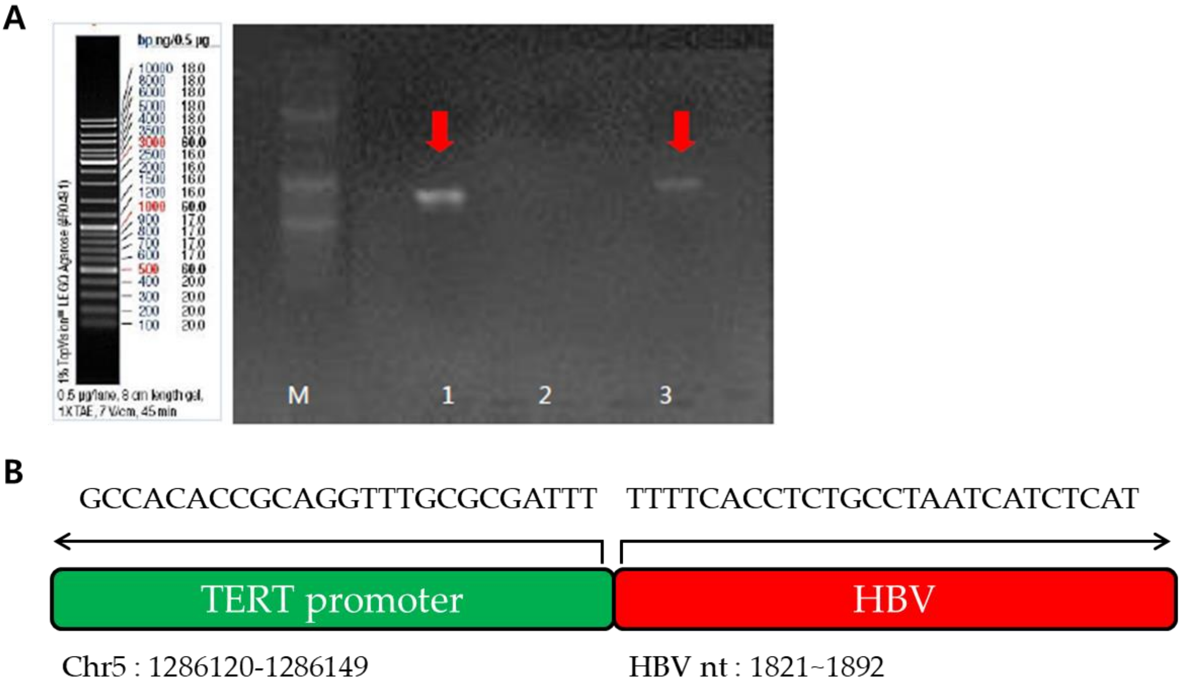

C

CTGGCGCCACACCGCAGGTTTGCGCGATTTTTTTCACCTCTGCCTAATCATCT
CATGTTCATGTCCTACTGTTCAAGCCTCCAAGCTGTGCCTTGGGTGGC

Sanger sequencing validation of a representative case (Patient 275T). (**A**) Detection of HBV integration (arrows). (**B**) Schematic view of the chimeric junction between human DNA and HBV DNA. (**C**) Sequences on both sides of the junction between human DNA (TERT promoter) and HBV DNA insert (HBV genome nt 1,821-1,982). The HBV sequences are underlined. HBV, hepatitis B virus; TERT, telomerase reverse transcriptase.

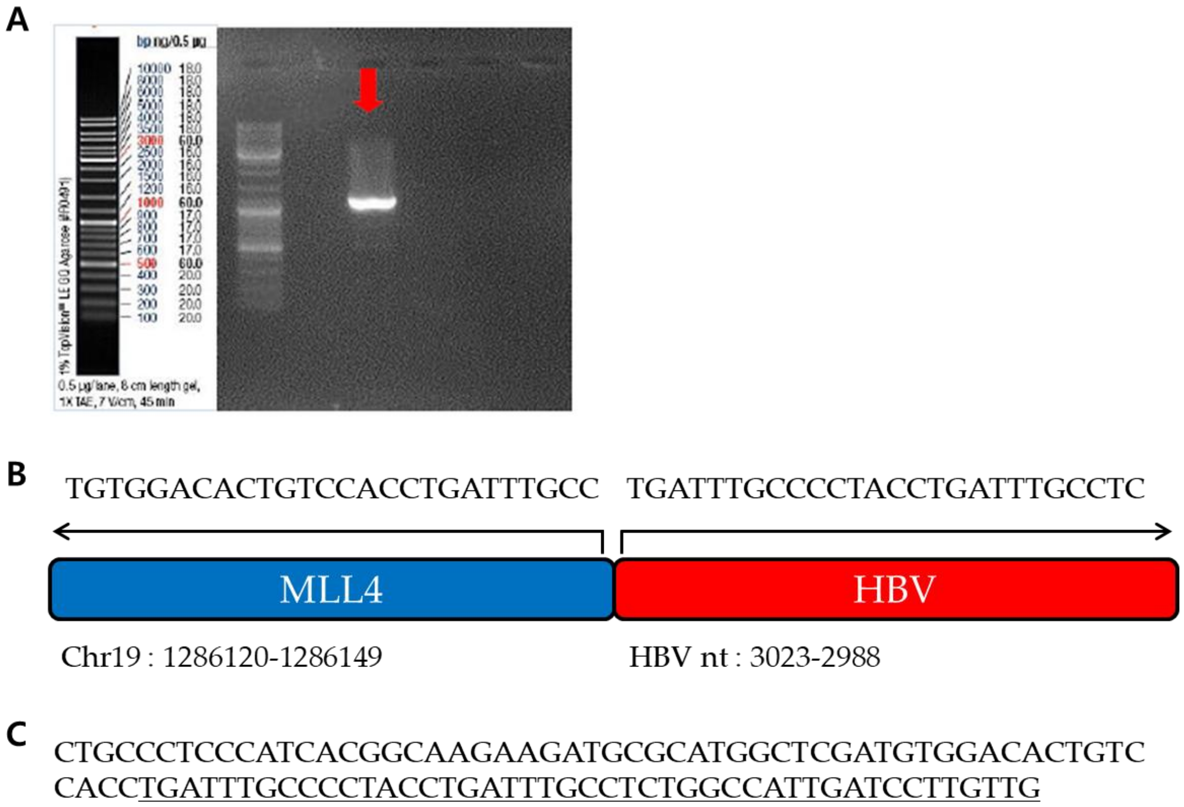

Sanger sequencing validation of a representative case (Patient 372T). (**A**) Detection of HBV integration (arrows). (**B**) Schematic view of the chimeric junction between human DNA and HBV DNA. (**C**) Sequences on both sides of the junction between human DNA (MLL4) and HBV DNA insert (HBV genome nt 3,023-2,988). The HBV sequences are underlined. HBV, hepatitis B virus; MLL4, mixed lineage leukemia 4.

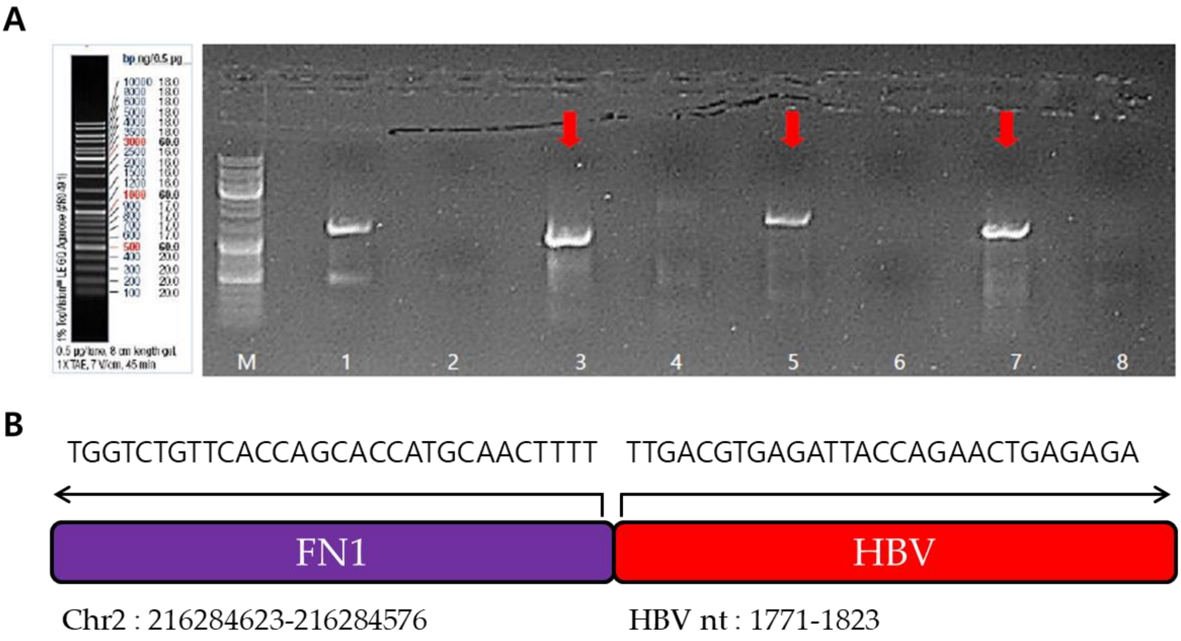

C

TACTAGGAGGCTGTAGGCATAAATTGGTCTGTTCACCAGCACCATGCAACTT
TTTTGACGTGAGATTACCAGAACTGAGAGAGAAGCCTGAGAGGTTTTCT

Sanger sequencing validation of a representative case (Patient 11A024). (**A**) Detection of HBV integration (arrows). (**B**) Schematic view of the chimeric junction between human DNA and HBV DNA. (**C**) Sequences on both sides of the junction between human DNA (FN1) and HBV DNA insert (HBV genome nt 1,771-1,823). The HBV sequences are underlined. HBV, hepatitis B virus; FN1, fibronectin 1.

Supplementary Table S1. Validation results for the HBV integration sites

Sample	Chromosome	Position	Count	Strand	Function	Gene name	HBV location	HBV domain	Sanger validation
6T	Chr 5	1285170	754	Plus	Promoter	TERT	58	DNA polymerase, surface antigen	Yes
6T	Chr 5	1295193	230	Plus	Promoter	TERT	2734	DNA polymerase	Yes
160T	Chr 5	1295233	38	Plus	Promoter	TERT	1818	X protein, precore/core	No
160T	Chr 5	1295200	3178	Plus	Promoter	TERT	267	DNA polymerase, surface antigen	Yes
275T	Chr 5	1284120	7134	Plus	Intron	TERT	1821	X protein, precore/core	Yes
275T	Chr 5	1292309	4830	Plus	Intron	TERT	345	DNA polymerase, surface antigen	Yes
432T	Chr 5	1295171	630	Plus	Promoter	TERT	1974	Precore/core	Yes
432T	Chr 5	1295227	222	Plus	Promoter	TERT	944	DNA polymerase	Yes
1648T	Chr 5	1295166	4691	Plus	Promoter	TERT	1918	Precore/core	Yes
1648T	Chr 5	1295264	4258	Plus	Promoter	TERT	1787	X protein	Yes
1660T	Chr 5	1295166	1	Minus	Promoter	TERT	1914	Precore/core	No
T-170810	Chr 5	1295250	577	Plus	Promoter	TERT	2612	DNA polymerase	Yes
T-170810	Chr 5	1295276	538	Plus	Promoter	TERT	259	DNA polymerase, surface antigen	Yes
T-180713	Chr 5	1295258	338	Plus	Promoter	TERT	1814	X protein, precore/core	Yes
387T	Chr 19	36213221	2585	Plus	Intron	MLL4	1268	DNA polymerase	Yes
387T	Chr 19	36213231	1478	Plus	Intron	MLL4	1620	DNA polymerase, X protein	Yes
372T	Chr 19	36213725	3863	Plus	Intron	MLL4	1580	DNA polymerase, X protein	Yes
T-181016	Chr 19	36212913	4	Minus	Intron	MLL4	1253	DNA polymerase	Yes
N-171219	Chr 2	216250045	21	Plus	Intron	FN1	334	DNA polymerase, surface antigen	Yes
N-12A046	Chr 2	216284623	10	Minus	Intron	FN1	1825	X protein, precore/core	Yes

Supplementary Table S2. Genes with recurrent HBV integrations

Gene	Description	Function
**Genes with recurrent HBV integrations in tumors**		
TERT	Telomerase reverse transcriptase	Role of nicotinic acetylcholine receptors in the regulation of apoptosis, telomeres, telomerase, cellular aging, and immortality
PREX2	Phosphatidylinositol-3,4,5-trisphosphate dependent Rac exchange factor 2	Role of Rac protein activation and PTEN inhibition<br>Involvement of G-protein coupled receptor signaling pathway, phosphatidylinositol 3-kinase signaling, Rho protein signal transduction, and GTPase activity
MLL4	Mixed-lineage leukemia 4	Regulation of histone H3 lysine 4 monomethylation, cell-cycle progression, and cell viability
SCFD2	Sec1 family domain containing 2	Regulation of cell proliferation and vesicle docking involved in exocytosis and protein transport
ADAM12	ADAM metallopeptidase domain 12	Role of EGF receptor transactivation<br>Regulation of cell-to-cell interaction
**Genes with recurrent HBV integrations in non-tumors**		
FN1	Fibronectin 1	Regulation of angiogenesis, protein phosphorylation, cell adhesion, calcium-independent cell-matrix adhesion, and leukocyte migration
DCC	DCC netrin 1 receptor	Involvement of nervous system
OAZ2	Ornithine decarboxylase antizyme 2	Regulation of cellular amino acid metabolic process, polyamine metabolic process, polyamine biosynthetic process, and intracellular protein transport
NPAT	Nuclear protein, coactivator of histone transcription	Regulation of cell cycle and coordinated transcriptional activation of multiple histone subtype<br>Role of immune responses modulation
GRIK4	Glutamate ionotropic receptor kainate type subunit 4	Involvement of glutamate receptor signaling pathway, chemical synaptic transmission, and ion transmembrane transport
ENOX1	Ecto-NOX disulfide-thiol exchanger 1	Involvement of rhythmic process, oxidation-reduction process, electron transport, and homologous recombination repair frequency
SNCAIP	Synuclein alpha interacting protein	Role of Parkin in the ubiquitin-proteasomal Pathway, dopamine metabolic process, and cellular protein metabolic process
ANO3	Anoctamin 3	Role of neuronal excitability modulation<br>Regulation of lipid transport, and calcium activated phospholipid scrambling

Supplementary Table S3. Mapping quality (MQ) and base call accuracy

MQ of phred score	Probability of incorrect base call	Base call accuracy
10	1 in 10	90%
20	1 in 100	99%
30	1 in 1000	99.9%
40	1 in 10000	99.99%

Supplementary Table S4. PCR condition and primer design for validation

Primers for TERT		
**Sample**	**F/R**	**Sequence**
6T	Forward	5′- GAGCCACCAGCACAAAGAG -3′
	Reverse	5′- TTCTGTTCCCAATCCTCTGG -3′
160T	Forward	5′- CACACAGAAACCACGGTCAC -3′
	Reverse	5′- GGCGAGGGAGTTCTTCTTCT -3′
275T	Forward	5′- TTCCAACTCCCAGGTTCAAG -3′
	Reverse	5′- GGCGAGGGAGTTCTTCTTCT -3′
432T	Forward	5′- GGTCGTTGACATTGCTGAGA -3′
	Reverse	5′- GCCTGAGAACCTGCAAAGAG -3′
1648T	Forward	5'- GAGCCACCAGCACAAAGAG -3′
	Reverse	5'- ATCGTCCCCTTCTTCATCTG -3′
1660T	Forward	5'- AGGTCTTGCCCAAGCTCTTA -3′
	Reverse	5'- CTCCTTCAGGCAGGACACCT -3′
T-170810	Forward	5'- GTTGGCGAGAAAGTGAAAGC -3′
	Reverse	5'- GCCTGAGAACCTGCAAAGAG -3′
T-180713	Forward	5'- GAGCCACCAGCACAAAGAG -3'
	Reverse	5'- GCTTTCACTTTCTCGCCAAC -3'
Conditions		95℃ for 5min, 95℃ for 30sec, 58℃ for 30sec, 72℃ for 2min with 35 cycles, and 72℃ for 10 min

Primers for MLL4		
**Sample**	**F/R**	**Sequence**
387T	Forward	5′- AACCCTCAGAACCTGCCTTT -3′
	Reverse	5′- ACTACTGCCTCACCCATATCGT -3′
372T	Forward	5′- AGGAGAGAGGGAGCCAAGTC -3′
	Reverse	5′- TGTCCAGTCTCCCCACCTAC -3′
T-181016	Forward	5′- GCTTTCACTTTCTCGCCAAC -3′
	Reverse	5′- GCCACAAAGGTTCCACGC -3′
Conditions		95℃ for 5min, 95℃ for 30sec, 58℃ for 30sec, 72℃ for 2min with 35 cycles, and 72℃ for 10 min

Primers for FN1		
**Sample**	**F/R**	**Sequence**
N-171219	Forward	5′- ACTGCCTCACCCATATCGTC -3′
	Reverse	5′- TGGGCTTTCAGGTTATCCAC -3′
N-12A046	Forward	5′- GAACCTTTGTGGCTCCTCTG -3′
	Reverse	5′- CTGAATCCTGGCATTGGTCT -3′
Conditions		95℃ for 5min, 95℃ for 30sec, 60℃ for 30sec, 72℃ for 2min with 35 cycles, and 72℃ for 10 min
